# *SETD5* Gene Haploinsufficiency in Three Patients With Suspected KBG Syndrome

**DOI:** 10.3389/fneur.2020.00631

**Published:** 2020-07-24

**Authors:** Milena Crippa, Ilaria Bestetti, Silvia Maitz, Karin Weiss, Alice Spano, Maura Masciadri, Sarah Smithson, Lidia Larizza, Karen Low, Lior Cohen, Palma Finelli

**Affiliations:** ^1^Research Laboratory of Medical Cytogenetics and Molecular Genetics, IRCCS Istituto Auxologico Italiano, Milan, Italy; ^2^Department of Medical Biotechnology and Translational Medicine, University of Milan, Milan, Italy; ^3^Clinical Pediatric Genetic Unit, Pediatric Clinic, Fondazione MBBM, San Gerardo Hospital, Monza, Italy; ^4^The Genetics Institute, Rambam Health Care Campus, Haifa, Israel; ^5^Medical Cytogenetics and Molecular Genetics Laboratory, IRCCS Istituto Auxologico Italiano, Milan, Italy; ^6^Clinical Genetics, St. Michael's Hospital, University Hospitals NHS Trust, Bristol, United Kingdom; ^7^Genetics Unit, Barzilai University Medical Center, Ashkelon, Israel; ^8^Faculty of Health Sciences, Ben-Gurion University of the Negev, Be'er Sheva, Israel

**Keywords:** MRD23, 3p25 microdeletion syndrome, KBG syndrome, *SETD5* haploinsufficiency, WES

## Abstract

Mendelian disorders of the epigenetic machinery (MDEMs), also named chromatin modifying disorders, are a broad group of neurodevelopmental disorders, caused by mutations in functionally related chromatin genes. Mental retardation autosomal dominant 23 (MRD23) syndrome, due to *SETD5* gene mutations, falls into this group of disorders. KBG syndrome, caused by *ANKRD11* gene haploinsufficiency, is a chromatin related syndrome not formally belonging to this category. We performed high resolution array CGH and trio-based WES on three molecularly unsolved patients with an initial KBGS clinical diagnosis. A *de novo* deletion of 116 kb partially involving *SETD5* and two *de novo* frameshift variants in *SETD5* were identified in the patients. The clinical re-evaluation of the patients was consistent with the molecular findings, though still compatible with KBGS due to overlapping phenotypic features of KBGS and MRD23. Careful detailed expert phenotyping ascertained some facial and physical features that were consistent with MRD23 rather than KBGS. Our results provide further examples that loss-of-function pathogenic variants in genes encoding factors shaping the epigenetic landscape, lead to a wide phenotypic range with significant clinical overlap. We recommend that clinicians consider *SETD5* gene haploinsufficiency in the differential diagnosis of KBGS. Due to overlap of clinical features, careful and detailed phenotyping is important and a large gene panel approach is recommended in the diagnostic workup of patients with a clinical suspicion of KBGS.

## Introduction

Mendelian disorders of the epigenetic machinery (MDEMs), also known as chromatin modifying disorders, are an expanding group of neurodevelopmental disorders (NDDs), caused by mutations in functionally related chromatin regulators (1). These syndromes are characterized by a significant phenotypic overlap as they share several clinical features including intellectual disability, developmental delay, growth retardation, and similar facial dysmorphisms (2). Interestingly, the phenotypic overlap between these syndromes appears to reflect the molecular interaction of chromatin modifiers, acting in common cellular mechanisms and pathways (3). Over recent years, genome wide analyses including chromosome microarray (CMA) and whole exome sequencing (WES) have been successfully utilized for this patient group to optimize molecular diagnosis and to refine the clinical phenotyping (4), resulting in MDEMs emerging as one of the most rapidly expanding groups (1).

KBG syndrome (KBGS, OMIM#148050), although functionally related, does not formally belong to the MDEMs group, while the mental retardation autosomal dominant 23 syndrome (MRD23, OMIM#615761) falls into this broad group of disorders. They are caused by *ANKRD11* (*Ankyrin Repeat Domain-Containing Protein 11*, OMIM^*^611192) gene haploinsufficiency and *SETD5* (*Set Domain-Containing Protein 5*, OMIM^*^615743) gene loss-of-function (LoF) mutations, respectively (5, 6).

KBGS is a disorder of chromatin-associated transcription machinery, mainly characterized by neurodevelopmental delay, macrodontia of the upper central incisors, facial dysmorphism, short stature, and skeletal anomalies (7). *ANKRD11*, located on chromosome 16q24.3, encodes a chromatin regulator of histone acetylation during neural development (8). The gene is also able to recruit both histone deacetylases (HDACs) and histone acetyltransferases (HATs) to promoters regulating the transcription of target genes (9).

The MRD23 phenotype is characterized by ID, facial dysmorphisms, skeletal anomalies, behavioral problems and speech and language difficulties (10). *SETD5*, located on chromosome 3p25, encodes for a putative methyltransferase, and belongs to the “writers” group of epigenetic ID genes (11). The gene is widely expressed across the brain, where it regulates gene transcription through its interaction with the histone deacetylase 3 (HDAC3) and RNA polymerase II associated factor 1 (PAF1) complexes (12). *SETD5* is also the strongest candidate gene of the 3p25 microdeletion syndrome, as patients harboring either heterozygous *SETD5* LoF mutations or 3p25 microdeletions spanning *SETD5* show an overlapping phenotype.

## Subjects and Methods

### Patients

In this study, we performed high resolution array CGH and trio-based WES on three previously molecularly unsolved patients with an initial suspected diagnosis of KBGS, in order to detect novel pathogenic variants. Patients underwent clinical phenotyping by their referring clinical geneticists, and according to the recently proposed diagnostic aid for KBG syndrome (13), were compatible with a clinical KBGS diagnosis. This study was approved by the ethical committee of IRCSS Istituto Auxologico Italiano, and written informed consent was obtained for each subject and/or their parents.

### High Resolution Array Comparative Genomic Hybridization (a-CGH) Analysis

High resolution array comparative genomic hybridization (a-CGH) analysis was performed on genomic blood DNA of P1, P2, and their parents, using the SurePrint G3 Human CGH Microarray Kit 2 × 400 K in accordance with the manufacturer's instructions (Agilent Technologies, Palo Alto, CA). Data extraction and analysis were performed using Agilent CytoGenomics v.3.0 (Agilent Technologies, Palo Alto, CA). Detected copy number variants (CNVs) were classified according to guidelines reported by Miller et al. (14) and by the American College of Medical Genetics (15).

### Whole Exome Sequencing

For P2, WES was performed on 50 ng of genomic DNA of each member of the trio (index patient and parents), prepared using the Agilent SureSelect Clinical Research Exome enrichment kit (Agilent, Santa Clara, California) that enables the enrichment of the exonic regions and the splice sites flanking regions of all human exons, followed by sequencing with 150-bp paired-end reads on the NextSeq500 platform (Illumina, San Diego, California).

Reads were mapped to the human genome (hg 19/GRCh37) using the Burrows-Wheeler Alignment tool (16). The variant calling was performed with the GATK Haplotype Caller (Broad Institute, Cambridge, MA) (17). The resulting variant call format (VCF) files were annotated using the Web ANNOtate VARiation (wANNOVAR) software (18).

Given the rarity of the suspected disease, the variant list was filtered according to an allele frequency ≤ 0.1% according to gnomAD (http://gnomad.broadinstitute.org/), the ExAC Browser of Broad Institute (19), and 1000 Genomes database. Variants were sorted out following both the *de novo* dominant and the autosomal recessive inheritance models; the functional impact, the bioinformatics predictions, and the presence in OMIM genes were further considered for variants analysis.

For P3, Trio exome sequencing was performed in the context of a pilot project funded by the Israeli ministry of health. Sequencing was performed in a CLIA certified laboratory (Gene by gene, Huston, TX, USA), on a Novaseq6500 platform (Illumina, San Diego, California) using the Twist Human Core Exome Kit (Twist, San Francisco, California). Mapping of the obtained reads to the reference genome (build GRCh37/hg19), variant calling, annotation, and data analysis were done using the Genoox data analysis platform Ltd. (Genoox, Palo Alto, California). We filtered sequencing data on a trio-based paradigm to identify recessive (homozygous and compound heterozygous), X-linked, and potential *de novo* variants in the proband. Variants were prioritized based on their effect on the protein and minor allele frequency <1% in general population databases, such as gnomAD, the Greater Middle-East Variome (http://igm.ucsd.edu/gme/), and the Rambam Genetics Institute internal database of over 1,500 Israeli exomes.

Variant clinical classification was performed according to ACMG classification (20) using the varsome database (21).

### Sanger Sequencing

The pathogenic variants identified by WES analysis were validated by Sanger Sequencing using the Big-Dye® Terminator v3.1 Cycle Sequencing Kit (Thermo Fisher Scientific) and the Applied Biosystems Abi Prism 3500 Sequencer.

Sequences were then aligned to the human reference genome sequence (human genome assembly GRCh37/hg19) and analyzed with the ChromasPro 1.5 software (Technelysium Pty Ltd., Tewantin, QLD, Australia). Primers are available upon request.

## Results

**Figure 1:**
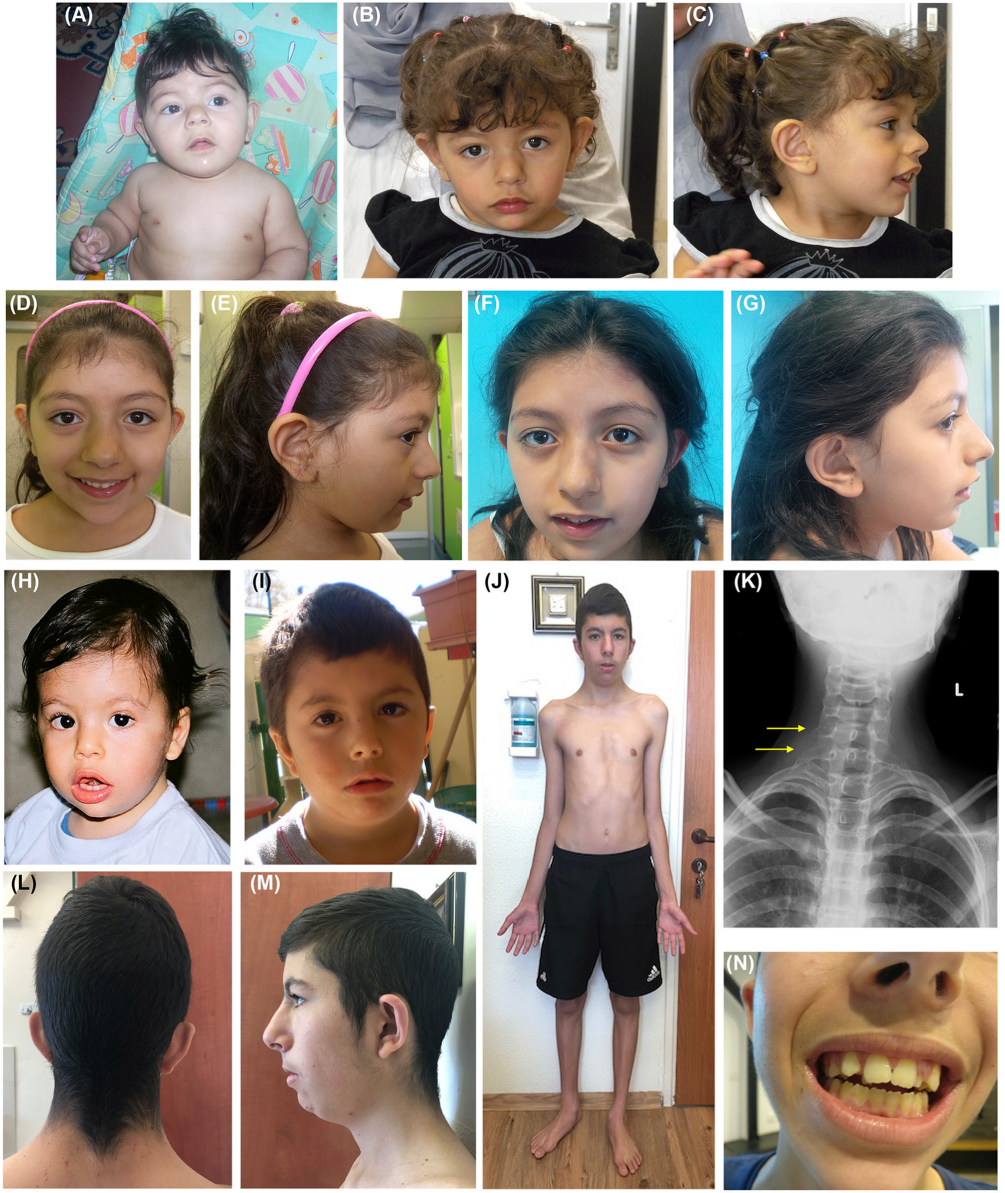
Clinical features of patients 2 and 3. **(A)** P2 as an infant and at the age of 2 **(B,C)**, 6 **(D,E)**, and 8 **(F,G)** years. **(A–G)** Facial dysmorphisms of P2 evolving with age include low anterior hairline, thick eyebrows, long palpebral fissures, and bulbous nose. **(H)** P3 at 12 months, and at the age of 3 **(I)** and 16 years **(J,L–N)**. **(H–J, L–N)** Facial dysmorphisms of P3 evolving with age include triangular face, low anterior, and posterior hairline **(L)**, synophris, long palpebral fissures, anteverted nares, long philtrum, thin upper lip, low set and overfolded ears, overcrowded, and misplaced teeth **(N)**. Other findings such as pectus excavatum, flat foot, and slender habitus can be noticed as well **(J)**. **(K)** P3 chest radiograph revealing accessory cervical ribs.

The clinical features of the three reported patients are detailed in Table 1. Facial appearances of patients 2 and 3 at evolving ages (from birth to the age at diagnosis) are shown in Figure 1.

**Table 1:** Clinical features of the three reported patients in comparison with previously reported patients with *SETD5* mutations/deletions.

**Clinical features**	**PT1**	**PT2**	**PT3**	**Previously reported cases (*n* = 32)^a^^,^^b^^,^^c^**	**Overall (*n* = 35)^**a**^**
Age at evaluation	12y8ms	11y6m	17y		
Sex	F	F	M		
Pathogenetic variant (GRCh37)	3p25.3(9387774_9503839) × 1 dn	c.959delA; p.Lys320AsnfsTer15	c.1573_1574delTT; p.Leu525ArgfsTer17		
**GROWTH**					
Height (cm)	146 (10th)	141.3 (10th−25th)	168 (25th)		
Weight (kg)	34 (3rd)	42.7 (50th)	45.8 (5th)		
OFC (cm)	50.5 (<3rd)	53.5 (50th−75th)	53 (3rd)		
Growth retardation	N	N	N	11/29 = 38%	11/32 = 34%
Microcephaly	Y	N	Y	0/28 = 0%	2/31 = 9.5%
Height at birth (cm)	47 (5th)	49 (30th)	na		
Weight at birth (kg)	2.570 (3rd)	3.490 (65th)	2.700 (5th)		
OFC at birth (cm)	32.5 (8th)	32.5 (8th)	na		
**NEUROLOGIC**					
Developmental delay	Y	Y	Y	26/30 = 87%	29/33 = 88%
Speech delay	na	na	N	23/29 = 79%	23/30 = 77%
Motor delay	na	Y	Y	20/29 = 69%	22/31 = 70%
Intellectual disability	Borderline/mild (IQ 70)	Borderline (GQ 80)	Borderline/mild (IQ 73)	28/30 = 93%	31/33 = 94%
Seizures	N	N	N	3/31 = 10%	3/34 = 9%
ASD	N	N	Y	8/28 = 28.5%	9/31 = 29%
Behavioral abnormalities	Short attention span, inhibition, immaturity	Short attention span, phobias	N	21/32 = 66%	23/35 = 66%
**CRANIOFACIAL DYSMORPHISM**					
Brachycephaly	N	N	N	4/25 = 16%	4/28 = 14%
Triangular face	Y	Y	Y	6/14 = 43%	9/17 = 53%
Long/smooth and/or prominent philtrum	Long	N	Long	19/28 = 68%	21/31 = 68%
Palatal defects	Y, narrow palate	N	N	4/26 = 15%	5/29 = 17%
Micrognathia	N	Y, mild	Y	9/23 = 39%	11/26 = 42%
Low-set/abnormal ears	Y	Y	Y	12/26 = 46%	15/29 = 52%
Hearing loss	Y	Y	N	2/22 = 9%	4/25 = 16%
Palpebral fissures	Long	Long	Long	8/23 = 35%	11/26 = 42%
Synophrys and eyebrow abnormalities	Large eyebrows	Broad bushy eyebrows, and mild synophrys	Broad bushy eyebrows, and synophrys	11/25 = 44%	14/28 = 50%
Ptosis	Y	N	N	3/25 = 12%	4/28 = 14%
Anteverted nares	Y	Y	Y	7/24 = 29%	10/27 = 37%
Broad/low nasal bridge and or abnormal shape	Broad and high prominent nasal bridge with bulbous nasal tip	Broad and high prominent nasal bridge with bulbous nasal tip	Broad and high prominent nasal bridge with bulbous nasal tip	21/26 = 81%	24/29 = 83%
Thin upper lip	Y	N	Y	10/16 = 62.5%	12/19 = 63%
Teeth anomalies	Macrodontia, ridged teeth, malposition of teeth	Macrodontia, ridged and crowded teeth, malposition of teeth	Macrodontia, ridged and crowded teeth, malposition of teeth	6/29 = 21%	9/32 = 28%
**OTHER ANOMALIES**					
Genitourinary defects	N	N	Cryptorchidism	5/22 = 23%	6/25 = 24%
Leg length discrepancy	N	N	N	3/19 = 16%	3/22 = 14%
Scoliosis, kyphosis, lordosis	na	N	Thoracic kyphosis	9/23 = 39%	10/25 = 40%
Hand anomalies	Mild brachydactyly	N	N	10/26 = 38%	11/29 = 38%
Postaxial polydactyly	N	N	N	7/25 = 28%	7/28 = 25%
Low hairline	Anterior	Anterior	Anterior and posterior	6/21 = 29 %	9/24 = 37.5%
Muscle hypotonia	na	na	Y, in infancy	10/18 = 55.5%	11/19 = 58%
Congenital heart disease	N	N	Y, mitral stenosis	11/28 = 39%	12/31 = 39%
Feeding difficulties	N	N	N	16/24 = 67%	16/27 = 59%
Gastrointestinal or abdominal wall anomalies	Mild constipation	N	N	8/22 = 36.5%	9/25 = 36%

a *Patients in which the presence or absence of each feature was not reported are not included*.

b *Rauch et al. (22), Grozeva et al. (6), Kuechler et al. (11), Kobayashi et al. (23), Szczałuba et al. (24), Parenti et al. (3), Green et al. (25), Powis et al. (10), and Aoi et al. (26)*.

c *Only patients with SETD5 loss of function mutations or intragenic deletions are considered*.

Patient 1 (Table 1) is the first born of non-consanguineous Italian healthy parents. She was born at term by cesarean section because of nuchal cord, after an uneventful pregnancy. She started walking at the age of 16 months and said her first words before 12 months. She had persistent mild constipation. She displayed mild intellectual disability (WISC-III: IQ = 70 at the age of 8 years and 9 months) and according to her parents demonstrated a short attention span. At the age of 9 a neuropsychological evaluation described an inhibited and immature personality, and she needed a support teacher at school. At 10 years old she was diagnosed with mild hearing impairment. She has not had any seizures.

Aged 12 her growth parameters were in the low normal range (height on the 25th centile, weight on the 3rd centile, and occipitofrontal circumference on the 10th centile). Her facial features are similar to KBGS patients, with triangular face, low hairline, thick eyebrows, broad nasal root and bulbous nasal tip, long philtrum, and broad incisors. She has long palpebral fissures with mild asymmetrical ptosis and mild strabismus. She wears glasses for astigmatism, and she has a nasal vocal quality.

Previous testing included standard karyotyping, *FMR1* analysis and *ANKRD11* sequencing which were all normal.

**Figure 2:**
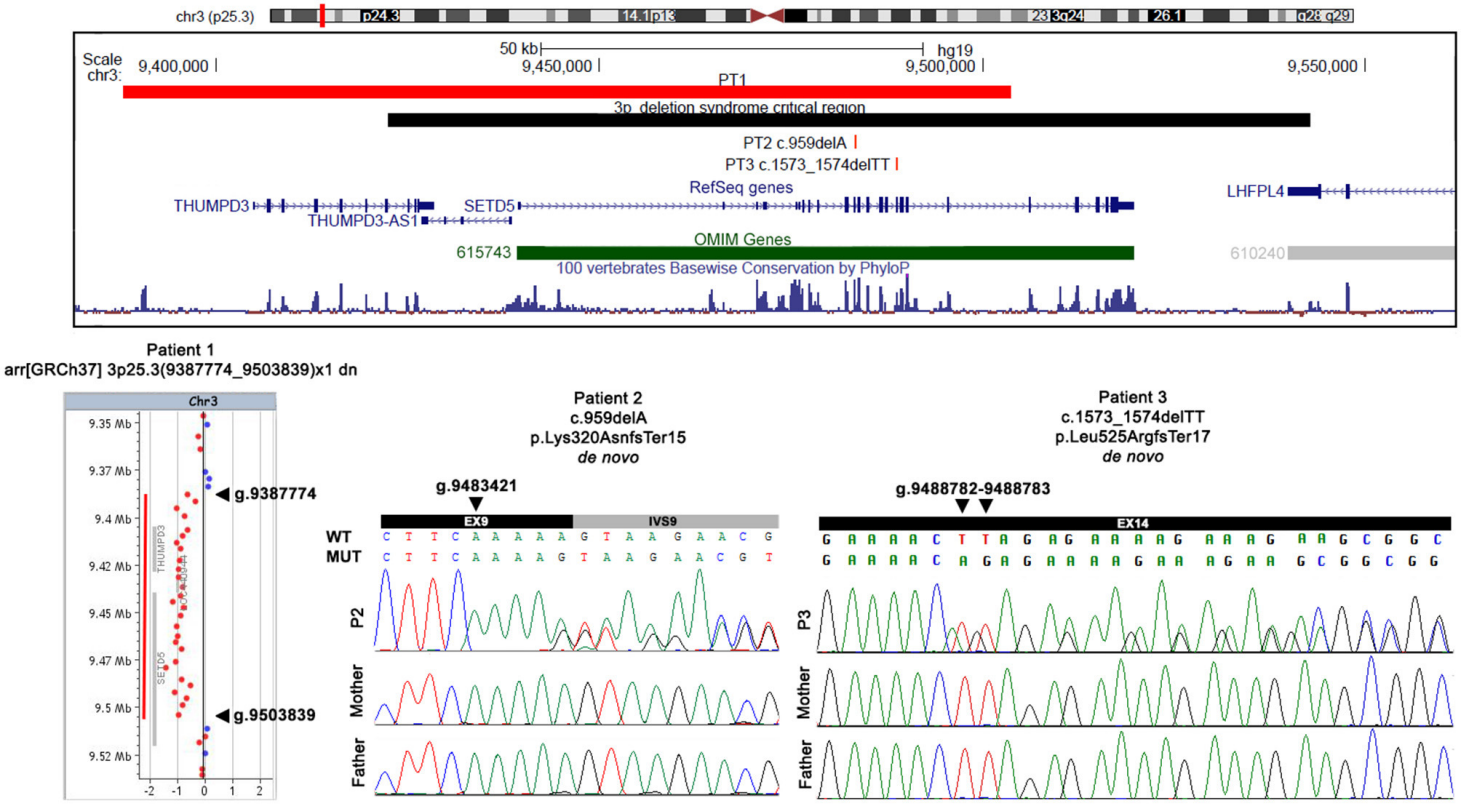
Physical map of the 3p25.3 genomic region and detected variants. In the upper part, the physical map of the 3p25.3 genomic region shows the deletion (red bar) detected in patient 1 and the two SNVs detected in patients 2 and 3. The 3pter-p25 deletion syndrome critical region is indicated by a black bar, the RefSeq genes are depicted in dark blue, and the OMIM disease genes in green. The image is a modification of a version obtained from the UCSC Genome Browser (human genome assembly GRCh37/hg19). In the lower part, an array CGH profile shows the 3p25.3 deletion identified in patient 1, and Sanger sequencing confirmation of the two SNVs detected in patient 2 and patient 3. Genomic positions refer to the human genome assembly GRCh37/hg19.

High resolution array CGH analysis detected a *de novo* 3p25.3 deletion of 116 kb (arr[GRCh37] 3p25.3(9387774_9503839) × 1 dn) involving three Refseq genes: *THUMPD3* (*Homo sapiens THUMP domain-containing 3*), *THUMPD3-AS1* (*THUMPD3 antisense RNA 1*), and *SETD5*, which is partially deleted as the proximal deletion breakpoint maps within intron 17 (transcript NM_001080517.2) (Figure 2). According to international guidelines this deletion was classified as pathogenic.

Patient 2 (Figure 1, Table 1) is the second born child of non-consanguineous Arab healthy parents. Delivery occurred by cesarean section at term after an uneventful pregnancy. Soon after birth, audiological screening revealed a conductive hearing impairment. Parents report she started sitting at 9 months of age, crawling at 17 months, and walking without support at the age of 19 months. Her first words started after 1 year of age, but of note she is bilingual (Italian-Arabic). Dental eruption occurred after 12 months. At the age of 15 months a development assessment was performed with Griffith's Scale showing a General Quotient of Development (GQ) = 86 and a significant deficit in motor abilities. A new assessment at the age of 27 months showed a GQ = 80.

We saw the patient for the first time when she was 2 years old. Physical examination revealed dysmorphic facial features including a low frontal and posterior hairline, thick eyebrows with mild synophrys, broad nasal root and bulbous nasal tip, long philtrum, and anteverted ears (Figures 1B,C). Her growth parameters were normal, and she has not had any seizures.

She had bilateral surgery at the age of 7 years because of a bilateral conductive hearing impairment and left stapes malformation, with apparently good results. She required surgical removal of deciduous teeth around 10 years old, because of dental crowding for lack of teeth loss. In the past she wore orthotic insoles for flat feet.

Patient 2 is now 11 years old and her growth parameters remain normal (10th−25th centile for height, 50th centile for weight and 50th−75th centile for occipitofrontal circumference). She attends school with a support teacher. No problems in sleep or behavior are reported. She wears glasses for myopia. Her facial appearance in many ways resembles the facial features seen in KBGS patients, including broad frontal incisors: as evident in Figure 1, bulbous nose and thick eyebrows present since infancy, with a progressively more evident large nasal root and prominent columella. Her decidual incisors are not notable, her permanent frontal incisors are large with accentuated mammelons.

Previous diagnostic testing included *NIPBL, SMC1A, SMC3, HDAC8, RAD21*, and *ANKRD11* sequencing, low resolution array-CGH and a *FMR1* test which were all negative as was a high-resolution array CGH.

Trio whole-exome sequencing (proband and parents) revealed a *de novo* frameshift deletion in *SETD5* (NM_001080517.2). The variant results in a single nucleotide deletion in exon 9 (c.959delA; p.Lys320AsnfsTer15) and is predicted to result in a premature stop codon after 15 amino acids (Figure 2). The alteration maps within the region encoding for the conserved protein SET domain.

Patient 3 (Figure 1, Table 1) is a 17-year-old male with mild intellectual disability (IQ = 73) and dysmorphic features.

He is the first born of non-consanguineous Sephardic Jewish parents. During pregnancy, standard karyotype analysis was performed by amniocentesis due to increased nuchal translucency (3.5 mm), confirming a normal male 46,XY chromosome picture. He was born at term with a birth weight of 2,700 g (5th centile). Hypotonia and global developmental delay were apparent from 5 months of age, and he started to walk at 30 months of age.

Bone age at 6 years was delayed by 2 years. Muscle biopsy (for a possibility of mitochondrial disorder) and head MRI at 7.5 years old were normal. Physical examination at 16 years of age (Figures 1J,L,M) noted that he has a triangular face, low anterior and posterior hairline, synophyrs, long palpebral fissures, epicanthal folds, low set overfolded ears, anteverted nares, long philtrum, thin upper lip, macrodontia (Figure 1N), overcrowded and misplaced teeth, pectus excavatum, genu varus, and flat foot. Chest radiograph revealed accessory cervical ribs (Figure 1K). As shown in Figure 1, the facial features became more evident with growth, in particular, the triangular face and overfolded ears were less apparent in infancy and childhood. Skeletal disproportion was confirmed by measurements performed at age 17: upper to lower segment ratio of 0.85, below the normal ratio (~1) for his age, and arm span to height ratio of 1.035 (normal range).

Previous genetic testing included low resolution chromosomal microarray, *ANKRD11* gene sequencing and NGS-based deletions/duplications analysis, and targeted NGS panel for Noonan genes. All previous analyses were negative.

A *de novo* 2 bp deletion in *SETD5* (NM_001080517.2) was detected by WES of the P3 trio. The variant maps within exon 14 (c.1573_1574delTT; p.Leu525ArgfsTer17) and is predicted to cause the formation of a premature stop codon after 17 amino acids (Figure 2).

The two identified point mutations are novel and according to ACMG classification, can be classified as pathogenic since they fulfill the PVS1, PS2, and PM2 criteria. Both variants have been confirmed by Sanger sequencing analysis (Figure 2).

## Discussion

With the advent of clinical exome sequencing, MDEMs have emerged as an increasingly expanding group of individually rare conditions showing partial phenotypic overlap, that collectively represent a relatively common cause of intellectual disability (1).

Here we report three patients with an initial clinical suspicion of KBGS, found to harbor *SETD5* genetic alterations, using integrated genome-wide analyses.

In all the three patients the pathogenic mechanism of the *SETD5* alteration is haploinsufficiency. Array CGH analyses identified in P1 a pathogenic deletion at 3p25.3, involving the *THUMPD3, SETD5*, and *THUMPD3-AS1* genes. The deletion overlaps the 3pter-p25 microdeletion syndrome critical region (OMIM#613792), confirming the crucial role of the above genes in the phenotype (Figure 2). WES was crucial to reach a definite molecular diagnosis in P2 and P3, both found to be carriers of an unreported *de novo* pathogenic variant within *SETD5*. Each variant introduces premature termination codons into the transcript which is expected to be either degraded by nonsense mediated decay (NMD) or translated into truncated dysfunctional proteins.

Our results confirm the importance of CMA and trio-based WES as diagnostic tools for molecularly unsolved patients.

Following the molecular results, clinical re-evaluation of the patients was compatible with the diagnosis of 3pter-p25 microdeletion syndrome (P1) and MRD23 (P2 and P3) (Table 1), respectively. Detailed cross-comparison of the overall phenotype of P1, P2, and P3 with the patients with MRD23 syndrome and small 3p25.3 microdeletions, described in the literature, pointed out that our three patients show the major clinical findings of MRD23 and 3pter-p25 microdeletion syndrome, namely ID, developmental delay, behavioral features, and facial dysmorphisms, in addition to minor features such as microcephaly, teeth anomalies, and hearing loss which have been rarely reported in previous patients (Table 1).

Taking into account the phenotype of our patients vs. that of already reported *ANKRD11* and *SETD5* mutated/deleted cases, a significant clinical overlap between KBGS, MRD23, and the chromosome 3pter-p25 deletion syndrome can be appreciated (Table S1). In particular, the cognitive profile ranked as mild intellectual disability, the developmental delay, and learning difficulties present in all three patients and Autism Spectrum disorder (ASD) in P3 and behavioral anomalies in P1 are mostly shared by all syndromes (Table 1, Table S1). Atrioventricular septal defects of patient 3, are described in all conditions as well. Of note, two out of the three patients do not show hand anomalies consistent with KBGS, which are good diagnostic clues for differential diagnosis (Table 1, Table S1). Regarding the facial appearance, the three patients have features that resemble KBGS. Previous large cohorts of KBGS described the facial features as not always being “gestalt” or reliable in some patients. In addition, KBGS expert review of P2 and P3's photographs did not conclude a satisfactory resemblance to KBGS in their facial features and, in addition for patient 3, with respect to his skeletal proportions. It is therefore important to recognize that whilst disorder specific diagnostic criteria have been proposed for individual conditions, these do have limitations in neurodevelopmental disorders with overlapping phenotypes. Therefore, it is important in such patients to undertake comprehensive genomic analyses and to then combine both the genotype and phenotype information together to reach an informed conclusion. In conditions such as these where doubt remains, expert opinion can be helpful in recognizing subtleties of the phenotype, which may not be apparent otherwise. This was true for our patients.

Currently, mutations in both *SETD5* and *ANKRD11* genes have been identified in patients with Cornelia de Lange (CdL) syndrome overlapping phenotypes, resulting in the terminology of “cohesin-related” syndromes (3, 26, 27). *ANKRD11* variants have also been associated with Coffin-Siris-like syndrome (28), another syndrome resulting from chromatin dysregulation, further underlining that mutations in different but functionally related chromatin-associated factors might result in strongly overlapping clinical pictures. Our study findings suggest that *SETD5* pathogenic variants, akin to what has already been observed in CdL-like phenotypes, can cause a phenotypic spectrum overlapping with KBGS. Interestingly, the ANKRD11 and SETD5 proteins physically interact at the molecular level as demonstrated by spectrometry assays performed in mouse embryonic stem cells and mouse neural progenitor cells (12). Moreover, both proteins interact with HDAC3, a key regulator of histone acetylation/deacetylation balance (8, 12).

Whilst there is significant phenotypic overlap between the above-mentioned conditions, however, they still remain distinct clinical entities. This is important for long term support to patients and families, as there are differences and subtleties in both clinical features and neurodevelopmental profiles between these conditions which become apparent only when patients are deeply phenotyped. “Lumping” conditions together is generally unhelpful and confusing for families and patients.

## Conclusions

In conclusion, the described patients confirm the increasing occurrence of genotype-driven syndrome recognition for subtle phenotypes of neurodevelopmental disorders caused by chromatin-related genes.

The description of our patients increases the number of reported cases with genomic alterations involving *SETD5*, providing novel molecular and clinical information.

We recommend that clinicians consider 3p25 microdeletion syndrome and/or MRD23 in the differential diagnosis of KBGS, and that *SETD5* should be included in cohesion-related gene panels in order to expedite molecular diagnoses in these patient groups.

## Data Availability Statement

The datasets presented in this study can be found in the ClinVar database (http://www.ncbi.nlm.nih.gov/clinvar/), accession numbers SCV001167679, SCV001167680, and SCV001167681.

## Ethics Statement

The studies involving human participants were reviewed and approved by the Ethical Committee of IRCSS Istituto Auxologico Italiano. Written informed consent to participate in this study was provided by the participants' legal guardian/next of kin. Written informed consent was obtained from the minor(s)' legal guardian/next of kin for the publication of any potentially identifiable images or data included in this article.

## Author Contributions

MC: study design, performed aCGH and WES experiments, analysis, interpretation of experimental and clinical data, and manuscript preparation. IB: performed WES experiment, WES data analysis, and variant validation. SM and AS: clinical data collection and interpretation. KW: WES data analysis and variant validation. MM: preliminary diagnostic testing by targeted NGS. SS: clinical data collection. LL: manuscript preparation and critical revision of the manuscript. KL: clinical data interpretation and critical revision of the manuscript. LC: clinical data collection and interpretation and critical revision of the manuscript. PF: study design, interpretation of experimental data, manuscript preparation, and critical revision of the manuscript. All authors read and approved the manuscript for submission.

## Conflict of Interest

The authors declare that the research was conducted in the absence of any commercial or financial relationships that could be construed as a potential conflict of interest.
